# Insanity in a Boy Four Years of Age, Following Fright

**Published:** 1846-02

**Authors:** 


*Insanity in a boy four years of age, following Fright.*—Dr. James Macdonald related a case of Insanity, occurring in a healthy boy, four years old. The first symptoms seemed to follow a fit of anger. He was refused permission to go into the country, and became very much excited. In the evening he was punished. This was followed by paroxysms of terror, the child having a frightened and startled look; sometimes he would become violent, striking at those around him, &c. This was in the latter part of June: there was heat of head, and the digestive organs were deranged. These latter were attended to, and then the shower-bath ordered; it however occasioned such terror that it was discontinued. A seton was placed in the back of the neck: some improvement followed, but the child again became worse, and gradually fell into a state of *idiocy*, passing urine and fæces involuntarily, losing the use of language, and acquiring a dull heavy look. The child was removed to the country, and on returning was attacked with intermittent fever of a tertian type. This was permitted to take its course, and continued till December. During its continuance, the mind gradually recovered its powers, so that by the middle of December the patient was entirely restored. There has been no instance of insanity in the family.—*Ibid.*

